# A novel UTMD system facilitating nucleic acid delivery into MDA-MB-231 cells

**DOI:** 10.1042/BSR20192573

**Published:** 2020-02-18

**Authors:** Hui Zhang, Yue Li, Fang Rao, Chun Liufu, Yi Wang, Zhiyi Chen

**Affiliations:** 1 Laboratory of Ultrasound Molecular Imaging, Department of Ultrasound Medicine, The Third Affiliated Hospital of Guangzhou Medical University, Guangzhou 510000, China; 2 Liwan Experimental Center, The Liwan Hospital of the Third Affiliated Hospital of Guangzhou Medical University, Guangzhou 510000, China

**Keywords:** gene delivery, miR-34a mimic, plasmid, UTMD

## Abstract

Gene therapy is emerging as a promising method for the treatment of various diseases. The safe and efficient delivery of therapeutic nucleic acids is a gene therapy prerequisite. Ultrasound, particularly in combination with microbubbles composed of biocompatible materials such as lipid, PLGA and chitosan, is a novel non-viral tool for gene transportation. Under ultrasound irradiation, microbubbles explode and generate pores in the cell membrane. Hence, genes can enter cells more easily. In order to transfect nucleic acids into MDA-MB-231 cells in a low-cost and non-viral manner for further breast cancer gene therapy studies, we explored ultrasound targeted microbubble destruction (UTMD) technology and evaluated the efficiency and safety of the delivery of plasmid encoding enhanced green fluorescent protein (pEGFP) and a microRNA-34a (miR-34a) mimic by UTMD. Sonovitro ultrasonic apparatus was employed to generate ultrasonic field, which was developed by our group. Ultrasonic parameters, including acoustic intensity (AI), exposure time (ET) and duty cycle (DC), were optimized at 0.6 W/cm^2^ AI, 20 s ET and 20% DC, the cell viability was not obviously impaired. Under these conditions, the UTMD-mediated transfection efficiency of pEGFP was greater than 40%. In addition to plasmid DNA, an miR-34a mimic was also successfully introduced into the cytoplasm by UTMD and found to inhibit proliferation, induce apoptosis of MDA-MB-231 cells and regulate downstream molecules. The present study indicates that further *in vivo* UTMD-mediated gene therapy studies are warranted.

## Introduction

With the rapid development of molecular biology, increasing number of gene mutations and alterations associated with diseases, such as cancer and inherited diseases, have been discovered. In addition, the capability to regulate or edit genes has been enhanced significantly. Gene therapy represents a promising means by which therapeutic genetic materials can be introduced into target cells to cure diseases [1]. In recent years, several preclinical or clinical gene therapy studies for the treatment of breast cancer have been launched [2,3]. Despite the great promise of gene therapy, the safe and efficient delivery of therapeutic genetic materials remains the foremost challenge to be overcome. Currently, viral vectors and non-viral vectors are available to transfect foreign nucleic acids into target cells *in vitro* or *in vivo*. Non-viral vectors, with the advantages of low cytotoxicity and immunogenicity, large gene payloads and ease of preparation, are increasingly being recognized as promising gene vehicles. However, low transfection efficiency poses one of the primary challenges for the extensive application of non-viral vectors [4,5].

Ultrasound has shown potential in promoting gene transfection efficiency *in vitro* and *in vivo* as indicated by a number of studies. To exert the maximal mechanical effect, ultrasound is commonly combined with acoustically responsive microbubbles or droplets [6]. Under ultrasonic irradiation, gas-filled microbubbles compress and expand cyclically and rapidly, resulting in microstreaming, cavitation and jetting. During this process, transient pores are generated in the cell membrane, and the permeability of the cell membrane is enhanced, which is conducive to gene introduction [7]. The technology, called ultrasound targeted microbubble destruction (UTMD), has been widely investigated for the delivery of drugs or genes to treat diseases such as solid tumors [8,9], obesity [10] and cardiovascular disease [11].

UTMD is a simple, time- and cost-effective technique, that can be accomplished by an ultrasound generator. Currently, the majority of ultrasound-mediated gene/drug delivery studies are performed by a clinical ultrasonic apparatus initially designed for diagnostic use. However, clinical instruments may not be suitable for fundamental UTMD research, in regard to the accuracy of the parameter setup and ease of manipulation, which are key factors for successful UTMD experiments. The safety, accuracy, reliability, flexibility and efficacy of UTMD has been dramatically enhanced with the emergence of ultrasonic instruments specifically designed for gene transfection or drug delivery. To the best of our knowledge, currently commercially available ultrasonic gene transfection systems include the Sonidel SP100 (Sonidel Ltd., Dublin, Ireland) [12], SonoPore KTAC4000 (Nepa Gene Co., Ltd., Chiba, Japan) [13] and Sonitron 1000 and 2000 (Richmar, Inola, OK, U.S.A.) [14]; each product has its own advantages. However, along with the urgent need for ultrasound-mediated gene/drug delivery studies, the number of professional ultrasound transfection apparatuses seems limited. To meet this need, our group has developed an ultrasonic transfection instrument called Sonovitro, which is specifically designed for *in vitro* gene transfection. The instrument is composed of a fixed ultrasonic probe, a water tank and a control panel by which UTMD parameters, including acoustic intensity (AI), duty cycle (DC) and exposure time (ET), can be accurately tuned. It has been demonstrated that AI, DC and ET are essential for the efficiency and safety of UTMD-mediated gene transfection [15].

In addition to the ultrasonic parameters, the types of microbubbles and genetic material to be transfected have an important influence on UTMD efficiency. Microbubbles responsive to ultrasound are mostly composed of biocompatible materials such as albumin, chitosan, PLGA and lipids, among which lipids are the most widely utilized [8]. In a previous study, we constructed a chitosan-conjugated anionic lipid microbubble and systematically optimized ultrasound parameters or efficient UTMD-mediated gene transfection. By UTMD technology, plasmid DNA could be efficiently delivered into HEK293T cells under the optimal conditions (AI 1.0 W/cm^2^, DC 10%, ET 60 s), the gene transfection efficiency was significantly enhanced, without obvious impairment of the cell viability [16]. However, the transfection efficiency is still not satisfactory. Compared with anionic microbubbles, cationic microbubbles (CMBs) are capable of electrostatically adsorbing nucleic acids, which is preferable for gene delivery [17]. Genetic material primarily refers to nucleic acids including plasmids and short nucleotides, such as short interfering RNA (siRNA) and microRNA (miRNA). Therapeutic genes are mostly transfected by UTMD in the form of plasmids due to the stability of plasmids. However, plasmids have some inherent limitations, such as low expression efficacy [18], resulting from their undesirable open circular and linear topological forms. In some cases, shorter nucleotides, such as miRNAs, may be preferred. MiRNAs are small non-encoding single-stranded RNAs comprising 21–25 nucleotides and can regulate multiple genes post-transcriptionally [19]. miRNAs have shown great potential in cancer treatment, among which microRNA-34a (miR-34a) has been the most widely investigated [20]. MiR-34a is down-regulated in breast cancer and plays an important role in tumor cell proliferation, invasion, migration, drug resistance and apoptosis [21]. MiR-34a generally functions by regulating downstream signaling pathways, such as Notch1 signaling, which is closely associated with tumorigenesis, lymph node metastasis and chemoresistance in human breast cancer [22]. However, in the course of miRNA application, low transfection efficiency, non-specificity and instability attenuate the efficacy of miRNAs [23]. UTMD provides a plausible solution.

In the present study, utilizing the gene transfection instrument we developed, ultrasound combined with cationic lipid microbubbles for nucleic acids, including a plasmid and miR-34a mimic, was systematically investigated *in vitro*. The cell line used in the present study was MDA-MB-231, which is representative of triple-negative breast cancer [24]. Our study aims to use UTMD to mediate efficient miRNA therapy for breast cancer and lays the foundation for further breast cancer gene therapy studies *in vivo*.

## Materials and methods

### Materials

DMEM basic medium and OPTI-MEM were from HyClone (Logan, UT, U.S.A.). Fetal bovine serum, trypsin and penicillin–streptomycin solutions were obtained from Life Technologies (Carlsbad, CA, U.S.A.). The MDA-MB-231 cell line was from ATCC (Manassas, VA). The cell counting kit-8 (CCK-8) was from Dojindo (Kumamoto, Japan). The plasmid encoding enhanced green fluorescent protein (pEGFP) was kept in our laboratory. The PureLink™ HiPure Plasmid Miniprep kit was purchased from Invitrogen (Carlsbad, U.S.A.). The miR-34a mimics, cy5-labeled miR-34a mimic and miRNA negative control (miR-NC) were synthesized by Genepharma (Shanghai, China). TRIzol reagent (RNAiso Plus) was purchased from Takara (Dalian, China). RevertAid Reverse Transcriptase was purchased from Thermo Fisher (Runcorn, Cheshire, U.K.). Primers were synthesized by Sagon Biotech (Shanghai, China). The 2× SGExcel FastSYBR mixture and miRNA qPCR master mix (SYBR Green) was purchased from Sangon Biotech (Shanghai, China). The Notch1, Hes1 and GAPDH antibodies were from OmnimAbs (CA, U.S.A.). CMBs (Uphere™ Trans+) were obtained from Trust Bio Sonics (Taiwan, China).

### Gene transfection by UTMD

**Figure 1:**
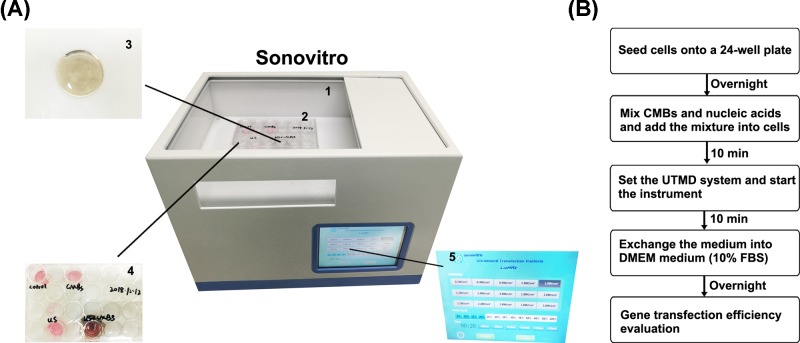
Illustration of the UTMD system (**A**) Configuration of the UTMD system. The Sonovitro apparatus is composed of a lid (1) a water tank (2), an ultrasonic probe (3) and a control panel (5). Cells incubated in a 24-well plate (4) were placed in the center of the probe. The area beneath the plate was filled with sterilized distilled water. (**B**) Flowchart of UTMD. The transfection procedure can be accomplished within 30 min.

The *in vitro* gene transfection system (Figure 1A) and UTMD flowchart (Figure 1B) are illustrated. The Sonovitro instrument was composed of a water tank, an ultrasonic probe and a control panel. The day before gene transfection, 1 × 10^5^ MDA-MB-231 cells/well were seeded and incubated in DMEM supplemented with 10% fetal calf serum in a 24-well plate. After 24 h, when the cell confluency reached approximately 80%, the medium was exchanged with 200 μl of serum-free OPTI-MEM medium. Concurrently, 10 μl of CMBs and 10 μg of pEGFP or 300 nM miRNA mimics were mixed and incubated for 10 min. Then, the mixture was added to the cells, and the medium was supplemented to a final volume of 500 μl. The plate was gently shaken and placed on the ultrasonic probe. The gap between the bottom of the plate and the probe was filled with sterilized distilled water. Parameters including AI, DC and ET were set, and the instrument was activated. Subsequently, the plate was incubated in a 5% CO_2_, 37°C incubator for 10 min, the medium was exchanged with 500 μl of fresh DMEM supplemented with 10% fetal calf serum, and the cells were incubated overnight. Cells without any treatment, treated with CMBs and treated with ultrasound were set as negative controls.

### Cell viability assay

A CCK-8 assay was performed to evaluate cell viability. Twenty-four hours after the ultrasonic treatment of MDA-MB-231 cells, CCK-8 reagent was added to the cells at a final concentration of 10% and the cultures were incubated in a 5% CO_2_, 37°C incubator for 2 h. The absorbance at 450 nm was measured by an ELx808 Absorbance Microplate Reader (Bio-Tek, U.S.A.). Cell viability (%) was calculated according to the following formula: cell viability (%) = (A_sample_ − A_blank_)/(A_control_ − A_blank_).

### Analysis of gene transfection efficiency

The UTMD-mediated gene transfection efficiency of pEGFP was first evaluated under a fluorescence microscope and then quantitatively determined by flow cytometry. Briefly, MDA-MB-231 cells were washed twice in PBS and harvested by trypsinization. The cells were collected by centrifugation at 1000×***g*** for 5 min. The pellet was resuspended in PBS. The percentage of EGFP-positive cells was analyzed by a Millipore Guava easyCyte™ flow cytometer (MA, U.S.A.).

### Scanning electron microscopy

The alterations in cell membrane morphology attributed by UTMD were observed by scanning electron microscopy (SEM). At different time points after UTMD (0, 5, 15, 30 and 60 min), cells were washed twice with PBS and fixed by with 3% glutaraldehyde. SEM analysis was performed by a HITACHI S-3000N Scanning Electron Microscope (Hitachi Ltd., Japan).

### Intracellular localization analysis of a cy5-labeled miR-34a mimic

MDA-MB-231 cells (1 × 10^5^ cells/well) were inoculated on a 24-well plate with glass coverslips at the bottom. The next day, cy5-labeled miR-34a mimics at a final concentration of 300 nM were transfected by UTMD. Twelve hours later, the glass coverslips were washed twice with PBS and fixed with 4% paraformaldehyde. After washing twice with PBS, the cells were stained with DAPI solution. The intracellular localization of the cy5-labeled miR-34a mimic was analyzed by a Pannoramic MIDI scanner (3DHISTECH, Budapest, Hungary).

### RT-qPCR

Total RNA was purified and reverse transcribed by an miR-34a-specific primer (GTCGTATCCAGTGCAGGGTCCGAGGTATTCGCACTGGATACGACAACAAC) and a U6-specific primer (AACGCTTCACGAATTTGCGT). *U6* was used as a reference gene. qPCR was performed to quantify miR-34a in MDA-MB-231 cells according to the manufacturer’s instructions. The primer pair for miR-34a was as follows: miR-34a-forward, GGGTGGCAGTGTCTTAGC; miR-34a-reverse, GTGCAGGTCCGAGGT. The primer pair for U6 was as follows: U6-forward, CTCGCTTCGGCAGCACA; U6-reverse, AACGCTTCACGAATTTGCGT. The relative expression of miR-34a was calculated according to the 2^−ΔΔ*C*_T_^ method.

### Cell proliferation assay

Cell proliferation was analyzed by the CCK-8 kit. Cells transduced with miR-34a mimic or miR-NC by UTMD were seeded in a 96-well plate at a cell density of 5 × 10^4^ cells/well. At 1, 2, 3 and 4 days after plating, CCK-8 reagent was added to the cells at a final concentration of 10% and the cells were incubated in a 5% CO_2_, 37°C incubator for 2 h. The absorbance at 450 nm was measured by an ELx808 Absorbance Microplate Reader (Bio-Tek, U.S.A.).

### Apoptosis assay

Apoptosis was evaluated by an Annexin V-FITC/Propidium Iodide (PI) cell apoptosis detection kit (KeyGEN BioTech, Jiangsu, China). MDA-MB-231 cells were collected and stained with PI and Annexin V for 15 min at 25°C away from light and analyzed by flow cytometry (Guava easyCyte™, Millipore, MA, U.S.A.).

### Western blot analysis

Cells were lysed and total proteins were extracted using RIPA buffer. The sample was separated by SDS/PAGE and transferred to a PVDF membrane. The Notch1 and Hes-1 primary antibodies were added. A rabbit IgG-HRP antibody was used as the secondary antibody. The protein expression levels of Notch1 and Hes-1 were normalized to the GAPDH level.

### Statistical analysis

Statistical analyses were performed using GraphPad Prism 5 software (GraphPad Software Inc., La Jolla, California). The data were expressed as the mean ± standard deviation. Every experiment was performed independently three times. Group comparisons were performed using Student’s *t* test. *P*<0.05 was considered indicative of a statistically significant difference.

## Results

### Effects of UMTD on cell viability

**Figure 2:**
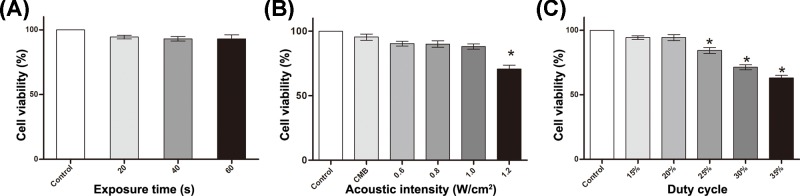
Cell viability analyses Ultrasound parameters included ET (**A**), AI (**B**) and DC (**C**). The data are represented as the mean ± SD (*n*=3). *P<0.05 compared with the control group.

The effects of ultrasound parameters on cell viability were investigated by CCK-8 assay. It allows very convenient assays by utilizing highly water-soluble tetrazolium salt WST-8, which produces a water-soluble formazan dye upon reduction in the presence of an electron mediator. The more formazan dye generated was proportional to the higher cells viability, so UTMD proliferation and toxicity analysis can be performed. When AI was fixed at 0.6 W/cm^2^, DC was fixed at 20% and 10 μl of CMBs were added, ET had no obvious effect on cell viability (Figure 2A). Because sonoporation is a transient physical process and CMCs were observed to explode completely within 20 s, ET was set 20 s in the subsequent experiments. Cell viability decreased significantly when AI was greater than 1.0 W/cm^2^, with a fixed 20% DC, 20 s ET and 10 μl of CMBs (Figure 2B). When AI was fixed at 0.6 W/cm^2^, and ET was fixed at 20 s, DC over 20% impaired obviously cell viability (Figure 2C). Finally, we chose 20 s ET, 0.6 W/cm^2^ AI and 20% DC as the optimal ultrasonic conditions in the subsequent UTMD studies.

### UMTD effect on cell membrane morphology and inducing apoptosis

**Figure 3:**
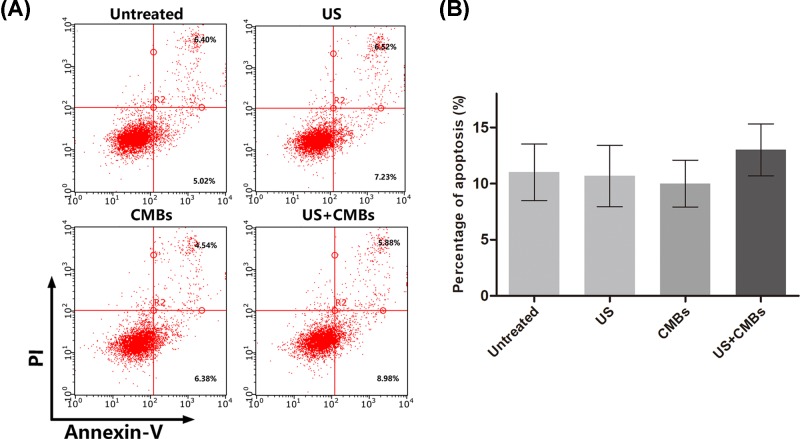
Apoptosis in MDA-MB-231 cells induced by different treatments (**A**) Apoptosis induction by US, CMBs and UTMD was determined and compared by Annexin V/PI staining and flow cytometry. (**B**) The apoptosis rate is presented as percentage of Annexin V-positive cells among the total cells. Data are presented as mean ± SD (*n*=3).

It has been reported that apoptosis is induced by therapeutic ultrasound and enhanced by the addition of contrast agents [25]. In this study, we evaluated the impact of UTMD using the conditions of 0.6 W/cm^2^ AI, 20% DC and 20 s ET on apoptosis by Annexin-V/PI staining and flow cytometry. In our UTMD system, compared with the untreated, US and CMBs groups, the US combined with CMB group showed no obvious induction of apoptosis (Figure 3A,B).

**Figure 4:**
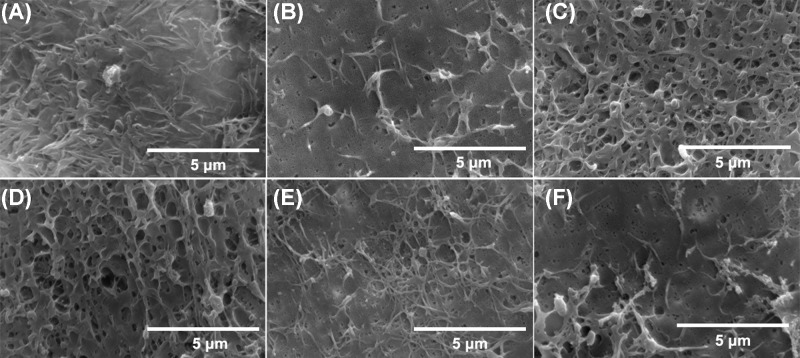
Cell membrane morphology analysis MDA-MB-231 cells untreated (**A**), cells 0 min after ultrasound treatment (**B**), and 0 min (**C**), 5 min (**D**), 15 min (**E**) and 30 min (**F**) after UTMD treatment were fixed with 3% glutaraldehyde and subjected to SEM analysis.

Efficient gene introduction mediated by UTMD is dependent on the so-called sonoporation effect induced by the interaction between ultrasound and microbubbles. SEM was performed to assess the membrane morphology alterations in our UTMD experiments. As shown in Figure 4, compared with the untreated group and the ultrasound-treated group, the UTMD group immediately displayed numerous nanoscale pores on the cell membrane in response to UTMD. As time passed, the cell membrane sealed, and the number of pores decreased. The cell membrane almost completely recovered after 30 min.

### Plasmid DNA transfection by UTMD

**Figure 5:**
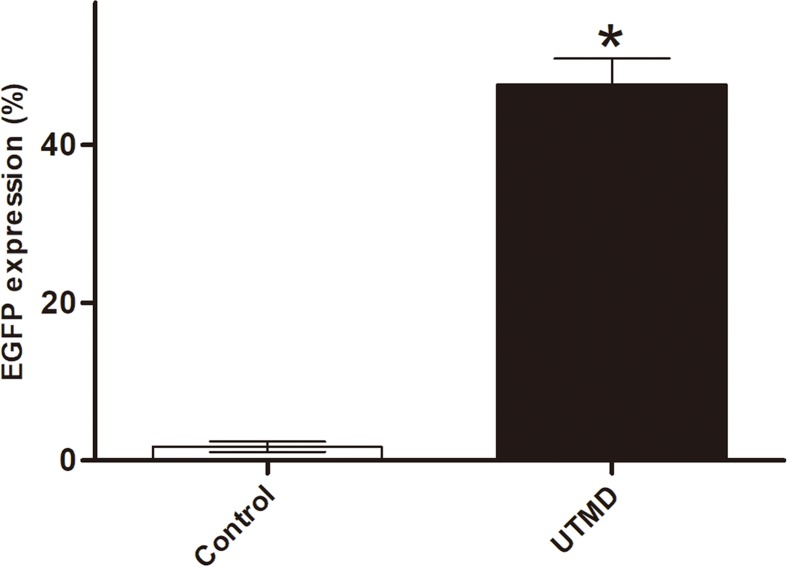
Transfection efficiency analysis The percentage of EGFP-positive cells was quantified by flow cytometry. Data are presented as the mean ± SD (*n*=3). *P<0.05 compared with the control group.

The amount of CMBs and plasmid DNA was first investigated to improve gene transfection efficiency. In our study, we obtained the optimal result when 10 μl of CMBs (approximately 4 × 10^5^ CMBs) and 30 μg plasmid were used in a 24-well plate. However, more CMBs or plasmid did not result in enhanced outcomes (data not shown). After UTMD treatment, the number of EGFP-positive cells increased significantly, and the transfection rate, which is represented by the percentage of EGFP-positive cells among the total cells, was greater than 40% by flow cytometry analysis (Figure 5). Ultrasound alone or CMBs alone had no obvious facilitatory impact on *in vitro* plasmid delivery (data not shown). Although CMB alone could introduce genes through chemical mechanisms, the procedure requires multiple cellular trafficking steps and takes hours [26]. In our study, CMBs-containing medium was exchanged with fresh medium within 10 min during which the chemical mechanism did not play a role in gene transduction. However, even though the protocol was performed according to the Lipofectamine™ 2000 protocol, the transfection efficiency of CMBs alone was limited and was much lower than that of UTMD (data not shown).

### MiR-34a mimic delivery by UTMD

**Figure 6:**
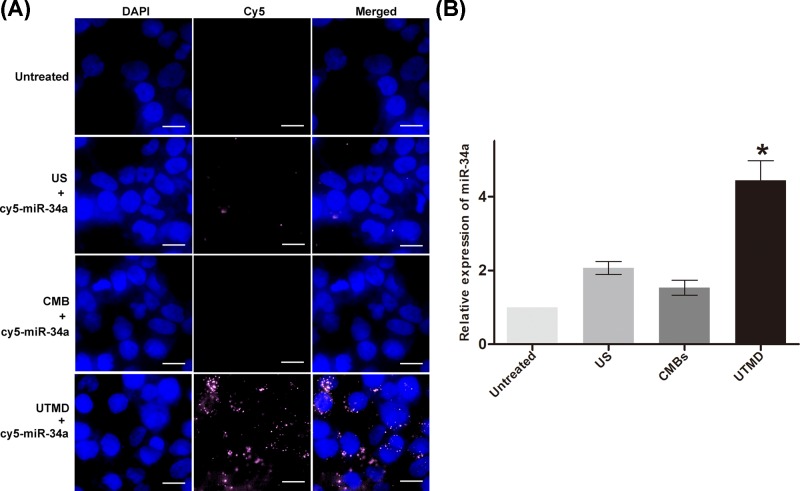
MiR-34a mimic localization analysis and quantification (**A**) Cellular uptake of a cy5-labeled miR-34a mimic (red) 24 h after different treatments. Cells left untreated, transfected with US + the miR-34a mimic and transfected with CMB + the miR-34a mimic were used as controls. Cells fixed on glass slides were stained with DAPI and visualized under a Pannoramic MIDI scanner. Cell nuclei were stained blue by DAPI. The scale bars are 20 μm. (**B**) Quantification of miR-34a by RT-qPCR. The miR-34a mimic transfected into MDA-MB-231 cells by UTMD was quantified by qRT-PCR. Data are representative of three independent experiments. *P<0.05, compared with other groups (untreated, US, CMBs, and UTMD).

It has been demonstrated that short nucleotides, such as miRNAs, are difficult to introduce by transfection reagents because of their limited negative charges and instability of the short nucleotide cationic polymer complexes [27]. In the present study, we synthesized an miR-34a mimic and asked whether the miR-34a mimic could be efficiently transfected into MDA-MB-231 cells by UTMD. The images in Figure 6A indicate that cy5-labeld miR-34a mimics (red) could enter cells with the aid of UTMD, rather than by ultrasound alone or CMBs alone. MiR-34a was further quantitatively assessed by RT-qPCR, and the UTMD group showed an approximately four-fold increase in miR-34a expression compared with that of the other groups (Figure 6B).

**Figure 7:**
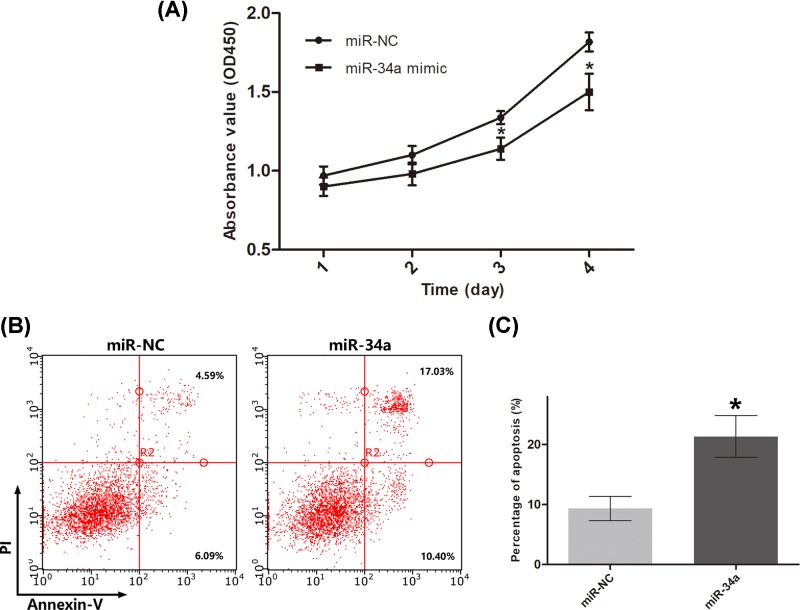
Analysis of proliferation and apoptosis of MDA-MB-231 cells (**A**) Inhibitory effect of miR-34a on the proliferation of MDA-MB-231 cells. (**B**) Apoptosis induced by miR-34a was assessed by Annexin V/PI staining and flow cytometry. (**C**) The apoptosis rate is presented as the percentage of Annexin V-positive cells among the total cells. The results are shown as the mean ± SD (*n*=3). *P<0.05, relative to the control group.

**Figure 8:**
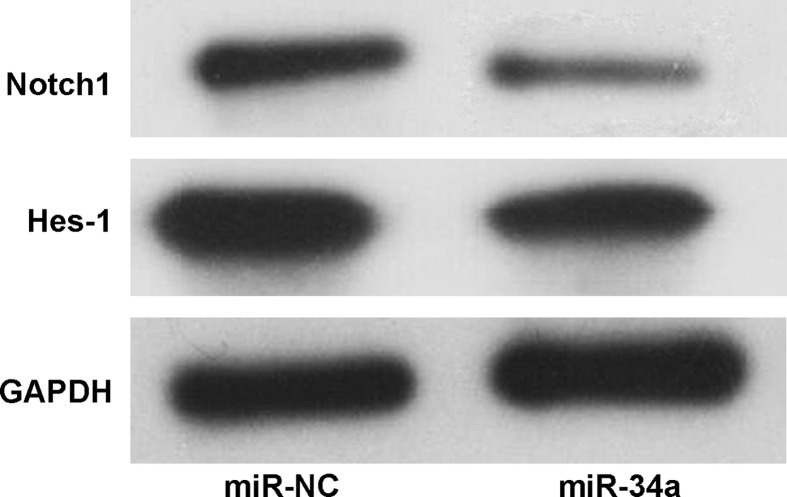
Analysis of Notch1 and Hes-1 expression in MDA-MB-231 cells Notch1 and Hes-1 protein expression were analyzed by Western blot 48 h after miRNA transfection by UTMD.

The function of the miR-34a mimic transfected by the UTMD system was further analyzed. A CCK-8 assay was carried out to investigate cell proliferation. As shown in Figure 7A, MDA-MB-231 cell proliferation was significantly inhibited in the miR-34a mimic group compared with that of the miR-NC group 3 days after UTMD-mediated transfection. Apoptosis induced by miR-34a was also evaluated by flow cytometry. As shown in Figure 7B,C, compared with the miR-NC group, the miR-34a mimic group displayed significantly increased apoptosis, with an apoptosis rate of approximately 25%. Finally, the expression of Notch1, which is a target of miR-34a, and its target Hes-1 was analyzed by Western blot. Both Notch1 and Hes-1 expression level were reduced by the miR-34a mimic (Figure 8).

## Discussion

As a simple and safe non-viral gene delivery method, UTMD has been widely explored in gene therapy applications for various diseases. Many mechanisms are involved in the process of UTMD-mediated gene transfection, and it is accepted that cavitation plays a key role [28]. During cavitation, a strong shock wave and microstream is produced, which generates nanoscale pores on the cell surface. As a result, the permeability of the cell membrane is enhanced and is conducive to gene introduction into the cells. Pores appear immediately after UTMD treatment, seal within 30 min and the morphology of the cells returns to normal, as shown in our study. The ultrasound parameters (AI, ET and DC) were optimized. Notably, AI over 1.0 W/cm^2^ resulted in obvious irreversible damage to the cells and cell viability decreased markedly (data not shown). Safety is the first thing to be considered in gene therapy studies *in vitro* or *in vivo*. Many factors, including ultrasonic parameters, microbubbles and gene transfection apparatuses, are associated with cell viability in *in vitro* UTMD experiments. In the present study, cell viability did not obviously decrease when ET was shorter than 60 s, with AI lower than 1.0 W/cm^2^ and DC lower than 20%. The ET was set to 20 s, and a longer ET did not help to improve the gene transfection efficiency. The microbubbles (USphere™) we use are commercially available and show biocompatibility comparable with that of clinical contrast. More importantly, the microbubbles are designed with a positive charge on the surface to absorb genetic material such as DNA or miRNA mimics, which can greatly facilitate ultrasound-mediated gene transfection [29]. In addition, CMBs can increase the attachment of bubbles to cells. Indeed, anionic microbubbles such as SonoVue [30] or albumin microbubbles [15] show reduced efficiency in UTMD gene transfection, necessitating the combination of other gene condensing positive components such as polyethylenimine (PEI) in our previous study [31].

To meet the urgent demand for UTMD studies, we developed an ultrasound transfection instrument—Sonovitro, which is specifically designed for *in vitro* applications. The apparatus is easy to operate, allows for the fine-tuning of parameters, including AI (0.2–3 W/cm^2^), ET (10 s) and DC (10–100%), and cannot be easily contaminated because it is equipped with a sterile compartment. To our knowledge, most of the ultrasonic devices we have mentioned above employ handheld probes, which could directly increase the instability of the experiment result. In contrast, the inner structure of Sonovitro consists of a fixed underneath probe and non-sound absorbing panel, ensuring the controllability of the experiment and reducing the interference of human factors.

The MDA-MB-231 cell line, which is relatively hard-to-transfect, was used to validate the instrument. Besides, gene transfection data from three other cell lines by Sonovitro were added as supplementary data for the present study, including HEK293, ovarian cancer cells (A2780) and ovarian cancer stem cells (OCSCs) (Supplementary Figure S1). Different cells have different tolerance to ultrasound, as well as transfection rate. We observed that a portion of HEK293 cells (adherent) floated after ultrasound irradiation, and the tumor cells had higher tolerance and transfection rates. Cancer stem cells, like stem cells, have the ability to self-renew and differentiate. The gene transfection rate of cancer stem cells by Sonovitro proved that UTMD-mediated gene transfection has a good performance in improving the permeability of different cell membranes, and better parameters need to be further explored according to cell types [32].

Based on systematic investigation of different factors, plasmid DNA was transfected into MDA-MB-231 cells to evaluate the efficiency of our UTMD system. By UTMD, pEGFP can be efficiently transfected into cells and express green fluorescent protein. The transfection rate reached more than 40% according to the flow cytometry results. Furthermore, miR-34a mimics were utilized to show the flexibility of the UTMD system. A number of studies have demonstrated that miRNAs play an essential role in tumor pathogenesis and development, and the regulation of the expression of miRNAs is a promising strategy for tumor treatment. In the present study, miR-34a mimics were transfected by our UTMD method to inhibit breast cancer cell proliferation and induce apoptosis at the cellular level. The results showed that miR-34a mimics delivered by UTMD efficiently inhibit MDA-MB-231 cell proliferation. In addition, downstream proteins including Notch1 and Hes-1, are down-regulated by miR-34a. Further studies will be directed toward therapeutic studies in animals.

In conclusion, we established a UTMD system for nucleic acid delivery into MDA-MB-231 cells and relevant factors and investigated. Both pEGFP and miR-34a mimics can be efficiently transfected by UTMD. The function of miR-34a delivered by UTMD was also investigated in MDA-MB-231 cells. The results show that the UTMD system has the potential for use in further *in vivo* gene therapy studies.

## Supplementary Material

Supplementary Figure S1

## Abbreviations

AI: acoustic intensity
CCK-8: cell counting kit-8
CMB: cationic microbubble
DC: duty cycle
ET: exposure time
pEGFP: plasmid encoding enhanced green fluorescent protein
PI: Propidium Iodide
SEM: scanning electron microscopy
US: Ultrasound
UTMD: ultrasound targeted microbubble destruction
