# Supplementary material for: A novel UTMD system facilitating nucleic acid delivery into MDA-MB-231 cells
Source: Biosci Rep. 2020 Feb 18;40(2):BSR20192573. doi: 10.1042/BSR20192573 (PMC7029150; doi:10.1042/BSR20192573)
Supplement: Supplementary Figure S1 [file BSR-2019-2573_supp.pdf]

As suggested by the reviewers, we added gene transfection data from three other cell lines, including HEK293, ovarian cancer cells (A2780) and ovarian cancer stem cells (OCSCs).

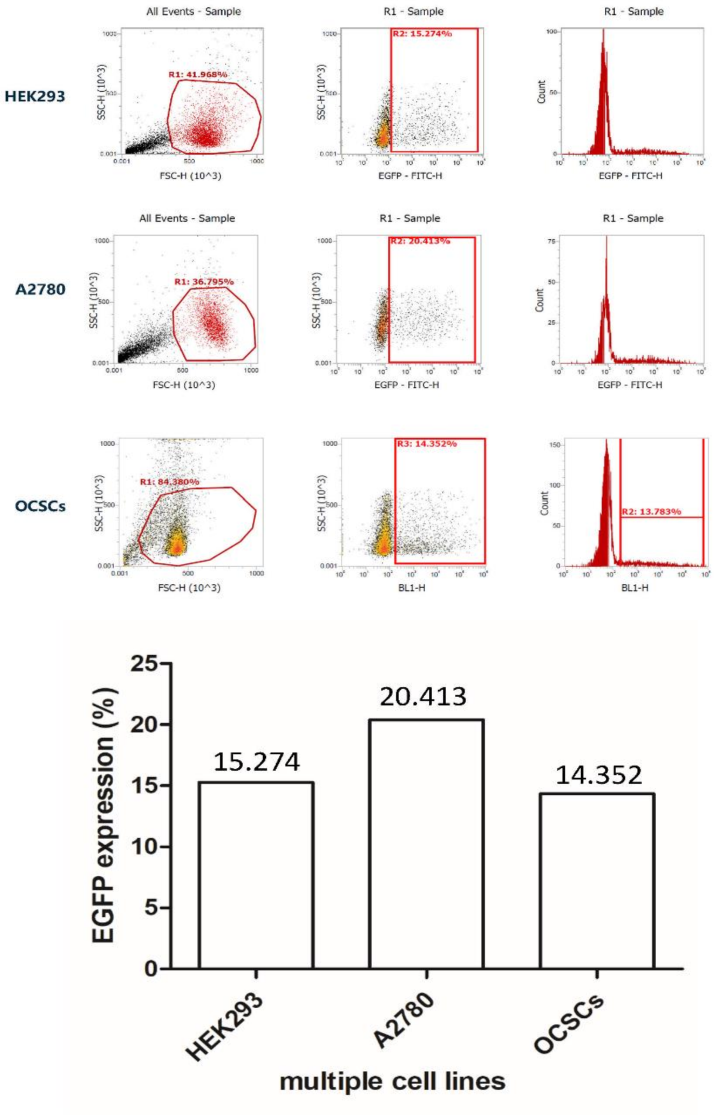

Figure S1. Quantitative results of gene expression in three cell lines transfected by using Sonovitro. All gene transfection of HEK293, ovarian cancer cells (A2780) and ovarian cancer stem cells (OCSCs) was performed through Sonovitro with the same parameters.
